# Specific effects of five subtypes of childhood maltreatment on suicide behaviours in Chinese adolescents: the moderating effect of sex and residence

**DOI:** 10.1017/S2045796023000604

**Published:** 2023-07-11

**Authors:** Chang Peng, Junhan Cheng, Fajuan Rong, Yan Wang, Yafei Tan, Yizhen Yu

**Affiliations:** 1 Department of Maternal, Child and Adolescent Health, School of Public Health, Tongji Medical College, Huazhong University of Science and Technology, Wuhan, Hubei, China; 2 Wuhan Children’s Hospital, Tongji Medical College, Huazhong University of Science and Technology, Wuhan, Hubei, China

**Keywords:** childhood maltreatment, emotional abuse, suicidal ideation, suicide attempts, suicide behaviours

## Abstract

**Aims:**

Although childhood maltreatment has been widely supported to be a robust predictor of suicide behaviours, the effects of different childhood maltreatment subtypes remain controversial and inconclusive. Moreover, whether the effects differ by sex in urban and rural adolescents is still unknown. This study aimed to quantify the associations between five subtypes of childhood maltreatment and different suicide behaviour involvement.

**Methods:**

A multistage cluster sampling method was adopted from April to December 2021 for adolescents aged 12 to 18 across five representative provinces of China. The Childhood Trauma Questionnaire-Short Form was used to measure childhood maltreatment subtypes. Suicide behaviour involvement was classified as none group, suicide ideator, suicide planner and suicide attempter. Confounding variables include demographic characteristics, smoking, drinking alcohol, depression and anxiety.

**Results:**

Among a total of 18,980 adolescents, 2021 (10.6%) were suicide ideator, 1595 (8.4%) were suicide planner and 1014 (5.3%) were suicide attempter. Rural females had the highest proportion of suicide ideator (13.8%) and suicide planner (11.5%). Multinomial logistic regression analysis indicated that five childhood maltreatment subtypes were independently associated with suicide behaviours, except for associations between sexual abuse and suicide ideator as well as suicide planner (*p* > 0.05). Moreover, these associations differ by sex and residence. After adjusted for interactions of different subtypes, structural equation model indicated that the direct effects of childhood maltreatment subtypes on suicide behaviours from high to low were emotional abuse (*β* = 0.363, *p* < 0.001), physical abuse (*β* = 0.100, *p* < 0.001) and sexual abuse (*β* = 0.033, *p* = 0.003), while the effects of physical neglect and emotional neglect were not significant (*p* > 0.05).

**Conclusions:**

Five subtypes of childhood maltreatment have specific and non-equivalence associations with suicide behaviours. Emotional abuse may have the strongest effect, and sexual abuse have an acute effect on suicide behaviours. Suicide prevention programs for Chinese adolescents could focus on those who experienced emotional, physical and sexual abuse. Furthermore, strategies should be tailored by sex and residence, and rural females deserve more attention.

## Introduction

Suicide is a fatal public health problem worldwide, and 1.3% overall mortality are the result of suicide in 2019 (Who, 2021). Particularly, 77% of these deaths happened in low- and middle-income countries. For adolescents, this proportion is up to 88%. In China, over 60,000 adolescents died by suicide in 2018 (Sun *et al.*, 2020). Therefore, it has great significance to explore risk factors of suicide for developing targeted prevention programs, especially for adolescents (Liu *et al.*, 2022; Turecki and Brent, 2016). To date, more scholars support that suicide is a developing process, which includes three stages of suicide behaviours before suicide death: suicide ideation (thoughts concerning ending one’s own life), suicide plans (plans about how to kill oneself) and suicide attempts (actual actions attempted to complete suicide) (Chen *et al.*, 2021b; Sveticic and De Leo, 2012). From this perspective, individuals involved in suicide behaviours or not could be classified into four groups: none group (without suicide ideation, plans and attempts), suicide ideator (having suicide ideation only, neither plans nor attempts), suicide planner (having suicide ideation and plans, but no attempts) and suicide attempter (having suicide ideation, plans and attempts concurrently) (Peng *et al.*, 2022).

Nowadays, a lot of prior work has supported that childhood maltreatment is a robust predictor of suicide behaviours (Chen *et al.*, 2021a; Grillault *et al.*, 2022; Stagaki *et al.*, 2022; Van Bentum *et al.*, 2022). Childhood maltreatment is typically defined as any action of commission or omission by parents or other caregivers that brings direct or potential harm, which generally encompasses five core subtypes: physical abuse, emotional abuse, sexual abuse, physical neglect and emotional neglect (Bernstein *et al.*, 2003). According to a recent meta-analysis, the pooled prevalence of five childhood maltreatment subtypes for Chinese primary and high school students from high to low is 47% (physical neglect) to 12% (sexual abuse) (Wang *et al.*, 2020).

Since both subtypes of childhood maltreatment and stages of suicide behaviours are not exclusive, disentanglement of the characteristics and pattern of the associations is warranted (Grillault *et al.*, 2022). Although many related previous studies have been conducted in community and clinical samples (Berardelli *et al.*, 2022; Grillault *et al.*, 2022; Hassan *et al.*, 2016; Walsh *et al.*, 2021; Xie *et al.*, 2018), several key research questions remain controversial and inconclusive. First, are the effects of different subtypes of childhood maltreatment on suicide behaviours equivalent or heterogeneous? Some prior literature supports that all forms of childhood maltreatment have positive impact on suicidality (Gong *et al.*, 2020; Lee *et al.*, 2021; Liu *et al.*, 2017; Miller *et al.*, 2013), while others indicate that not all childhood maltreatment subtypes could predict suicide behaviours (Behr Gomes Jardim *et al.*, 2018; Berardelli *et al.*, 2022; Hadland *et al.*, 2015; Hassan *et al.*, 2016; Zatti *et al.*, 2017). Second, for a certain childhood maltreatment subtype, are the effects on different involvement of suicide behaviours similar or specific (Liu *et al.*, 2017)? For example, a prior work reveals that frequent sexual abuse victims are more likely to attempt suicide rather than have suicide ideation or plans (Lee *et al.*, 2021). Third, which subtype of childhood maltreatment is the strongest risk factor of suicide behaviours? Some recent meta-analyses conclude that sexual abuse has stronger effect than other subtypes (Angelakis *et al.*, 2020, 2019). However, another meta-analysis demonstrates that emotional abuse could be the most robust predictor (Liu *et al.*, 2017). Fourth, whether the associations differ across sex and residence (Liu *et al.*, 2017; Mcmahon *et al.*, 2018; Salokangas *et al.*, 2019)? For example, some literature supports that adult females have higher prevalence of suicide ideation and suicide attempts than males, and rural China has three times higher rates of suicide than do urban China (Phillips *et al.*, 2002; Turecki and Brent, 2016). However, it is little known among Chinese adolescents.

To date, few previous studies have explored the specific impact of five childhood maltreatment subtypes on suicide ideation, plans and attempts between urban and rural adolescents in China. To fill the gaps, we conducted a nationwide study based on a large-size sample of Chinese adolescents across five representative provinces. The primary aim is to quantify the associations between five subtypes of childhood maltreatment (physical abuse, emotional abuse, sexual abuse, physical neglect and emotional neglect) and different suicide behaviour involvement (none group, suicide ideators, suicide planners and suicide attempters). Furthermore, the second aim is to examine the potential sex difference and residence difference in the associations between childhood maltreatment and suicide behaviours.

## Methods

### Study design and data collection

A multistage cluster sampling method was adopted from April to December 2021. In Stage 1, China was divided into five main geographic regions (eastern, southern, western, northern and central regions). Five provinces (Jiangsu, Guangdong, Yunnan, Gansu and Hubei) were randomly chosen in each region (Figure S1 in Supplementary material). In Stage 2, two cities were selected randomly in every selected province. In Stage 3, we selected one district in the urban areas and one county in the rural areas from each selected city. In Stage 4, one junior high school and one senior high school were selected randomly in each sample district or county. In Stage 5, we randomly chose four or six classes from each grade (7th to 12th) in each selected school based on enrolment size (Figure S2 in Supplementary material). Finally, all students in the selected class were invited to participate in the research voluntarily. Informed written consents were signed by every participant and their guardians before the filed survey.

#### Assessment

A custom-designed questionnaire was designed to collect demographic characteristics, high-risk health-related behaviours, suicide behaviours, childhood maltreatment and other psychological problems. Demographic variables included sex (male or female), residence (urban or rural), age (years old), grade (7th to 12th), family composition (living in a family with two biological parents, one biological parent or others), father and mother’s education level (primary school or less, junior high school, senior high school and college or more), family income (average family income per month in RMB, less than 1999, 2000–3999, 4000–5999, 6000–7999 and more than 8000). High-risk health-related behaviours included smoking (yes or no) and drinking alcohol (yes or no) (Turecki and Brent, 2016).

### Suicide behaviours

Suicide behaviours were measured based on three items regarding suicidality derived from the Global School–Based Student Health Survey (Ma *et al.*, 2021). Three stages of suicide behaviours were measured by the three following questions in the past year. Suicide ideation: “Have you ever seriously had thoughts of committing suicide?” Suicide plans: “Have you ever made a specific plan about how you would kill yourself?” Suicide attempts: “Have you ever tried to commit suicide?” The answer of these questions was defined as No (0 times) vs. Yes (1 to 4 times) (Lee *et al.*, 2021; Wan *et al.*, 2019). Due to some participants could simultaneously have suicide ideation, plans and (or) attempts, we classified all sample into four categories: None group (without any suicide behaviours), Suicide ideator (having suicide ideation only), Suicide planner (having suicide ideation and plans but no attempts) and Suicide attempter (having all suicide ideation, plans and attempts) (Sveticic and De Leo, 2012; Wang *et al.*, 2014).

### Childhood maltreatment subtypes

Childhood maltreatment subtypes were measured through the Childhood Trauma Questionnaire-Short Form (CTQ-SF) (Bernstein *et al.*, 2003), which contained five subscales: physical abuse, emotional abuse, sexual abuse, physical neglect and emotional neglect. Each subscale had five items, and each item was rated on a 5-point Likert scale (1 = never true, 2 = rare true, 3 = sometimes true, 4 = often true and 5 = always true). Therefore, the score of each subscale ranged from 5 to 25, and greater scores indicated more severe history of abuse or neglect. The Cronbach’s alpha coefficient of the CTQ-SF in the current study was 0.85.

### Other psychological problems

Other psychological problems included depression and anxiety. The nine-item Patient Health Questionnaire (PHQ-9) was used to measure major depression disorders (Kroenke *et al.*, 2001). Each item had a respond scored from 0 to 3. Total score ranged from 0 to 27, and a higher score of PHQ-9 indicated that the participant has more major depressive symptoms (Adewuya *et al.*, 2006). In the current study, the Cronbach’s alpha of PHQ-9 was 0.90. The seven-item Generalized Anxiety Disorder Scale (GAD-7) was used to screen generalized anxiety disorders (Spitzer *et al.*, 2006). Each item had a respond scored from 0 to 3. Therefore, total score of GAD-7 ranged from 0 to 21, and a higher total score showed that the participant has more generalized anxiety disorders (Lowe *et al.*, 2008). In this study, the Cronbach’s alpha for GAD-7 was 0.94.

#### Data analyses

First, demographic characteristics of sample and the proportion of suicide behaviour involvement were described by descriptive statistics. Continuous variables were depicted by mean (SD). Second, the chi-square test was used to compare the proportion of suicide behaviour involvement across different categories. ANOVA was used to compare the mean scores between different groups. Spearman’s correlation was used between two variables.

Third, to assess the associations between each subtype of childhood maltreatment and suicide behaviour involvement, a set of Multinomial logistic regression analysis was conducted for every subtype separately to estimate odds ratios (ORs) and 95% confidence intervals (95% CIs). Besides, to examine the influence of sex × residence in the associations, we conducted a subgroup analysis among urban males, urban females, rural males and rural females, separately. The significance level was set at *p* < 0.05, and all tests were two-sided. These analyses mentioned above were ran by SPSS 26.0.

Fourth, given potential multicollinearity between five subtypes of childhood maltreatment (Vachon *et al.*, 2015), Structural equation modelling was conducted to assess direct effects of five childhood maltreatment subtypes on suicide behaviours when we simultaneously entered all five subtypes into the model and adjusted the interaction between every two subtypes. Standardized *β* was used to estimate effect size. Structural equation modelling was ran by AMOS 21.0.

## Results

### Characteristics of participants

Of 21,207 respondents who sent back the questionnaire and provided the consent form, 831 were excluded because their age was over 18 or less than 12 years, 952 were excluded because they did not respond to any items regarding suicide behaviours (three items in total), 444 were excluded because the missing data of the whole questionnaire was over 15%. Finally, 18,980 participants’ questionnaires were qualified, and the actual valid response rate was 89.50% (18,980/21,207).

A total of 18,980 participants were almost equally distributed across sex (9472 males [49.9%] vs. 9508 females [50.1%]) and residence (9539 from urban areas [50.3%] vs. 9441 from rural areas [49.7%]). Their age ranged from 12 to 18, and mean (SD) of age was 14.98 (1.64). Other socio-demographic characteristics of the sample are presented in Table 1.

**Table 1.** General characteristics of participants and proportion of suicide behaviours

Variables	Total	Suicide behaviours				
		None	Suicide ideator	Suicide planner	Suicide attempter	*p-*value
Sex						<0.001
Male	9472 (49.9)	7727 (81.6)	783 (8.3)	594 (6.3)	368 (3.9)	
Female	9508 (50.1)	6623 (69.7)	1238 (13.0)	1001 (10.5)	646 (6.8)	
Residence						<0.001
Urban	9539 (50.3)	7386 (77.4)	923 (9.7)	709 (7.4)	521 (5.5)	
Rural	9441 (49.7)	6964 (73.8)	1098 (11.6)	886 (9.4)	493 (5.2)	
Age, M (SD)	14.98 (1.64)	14.99 (1.65)	15.09 (1.58)	15.00 (1.58)	14.64 (1.55)	<0.001
Grade						<0.001
7–9	10,033 (52.9)	7602 (75.8)	980 (9.8)	819 (8.2)	632 (6.3)	
10–12	8947 (47.1)	6748 (75.4)	1041 (11.6)	776 (8.7)	382 (4.3)	
Family composition						<0.001
Two biological parents	15,749 (83.0)	12,120 (77.0)	1656 (10.5)	1236 (7.8)	737 (4.7)	
Single biological parent	2144 (11.3)	1476 (68.8)	240 (11.2)	248 (11.6)	180 (8.4)	
Others	1017 (5.4)	690 (68.7)	119 (11.7)	105 (10.3)	94 (9.2)	
Missing data	70 (0.4)	–	–	–	–	
Father’s education						0.173
Primary school or less	1828 (9.6)	1366 (74.7)	186 (10.2)	170 (9.3)	106 (5.8)	
Junior high school	7191 (37.9)	5479 (76.2)	765 (10.6)	586 (8.1)	361 (5.0)	
Senior high school	5452 (28.7)	4148 (76.1)	560 (10.3)	435 (8.0)	309 (5.7)	
College or more	4368 (23.0)	3250 (74.4)	494 (11.3)	392 (9.0)	232 (5.3)	
Missing data	141 (0.7)	–	–	–	–	
Mother’s education						0.070
Primary school or less	3167 (16.7)	2364 (74.6)	361 (11.4)	280 (8.8)	162 (5.1)	
Junior high school	7140 (37.6)	5493 (76.9)	701 (9.8)	569 (8.0)	377 (5.3)	
Senior high school	4870 (25.7)	3675 (75.5)	519 (10.7)	413 (8.5)	263 (5.4)	
College or more	3621 (19.1)	2685 (74.2)	420 (11.6)	315 (8.7)	201 (5.6)	
Missing data	182 (1.0)	–	–	–	–	
Family income (RMB)						0.001
8000 ∼	4540 (23.9)	3467 (76.4)	470 (10.4)	339 (7.5)	264 (5.8)	
6000–7999	3479 (18.3)	2676 (76.9)	327 (9.4)	296 (8.5)	180 (5.2)	
4000–5999	4584 (24.2)	3497 (76.3)	477 (10.4)	372 (8.1)	238 (5.2)	
2000–3999	3879 (20.4)	2862 (73.8)	468 (12.1)	347 (8.9)	202 (5.2)	
∼1999	2145 (11.3)	1574 (73.4)	247 (11.5)	209 (9.7)	115 (5.4)	
Missing data	353 (1.9)	–	–	–	–	
Smoking						<0.001
Yes	671 (3.5)	400 (59.6)	66 (9.8)	95 (14.2)	110 (16.4)	
No	18,215 (96.0)	13,878 (76.2)	1949 (10.7)	1495 (8.2)	893 (4.9)	
Missing data	94 (0.5)	–	–	–	–	
Drinking alcohol						<0.001
Yes	1582 (8.3)	941 (59.5)	224 (14.2)	210 (13.3)	207 (13.1)	
No	17,291 (91.1)	13,327 (77.1)	1790 (10.4)	1378 (8.0)	796 (4.6)	
Missing data	107 (0.6)	–	–	–	–	
Childhood maltreatment						
Physical abuse, M (SD)	5.73 (1.84)	5.55 (1.58)	5.90 (1.84)	6.26 (2.36)	7.02 (3.15)	<0.001
Emotional abuse, M (SD)	6.72 (2.55)	6.22 (2.01)	7.55 (2.67)	8.35 (3.36)	9.48 (4.00)	<0.001
Sexual abuse, M (SD)	5.31 (1.43)	5.26 (1.30)	5.33 (1.30)	5.38 (1.35)	5.93 (2.77)	<0.001
Physical neglect, M (SD)	7.45 (2.90)	7.18 (2.75)	7.70 (2.90)	8.35 (3.15)	9.23 (3.49)	<0.001
Emotional neglect, M (SD)	8.89 (4.47)	8.25 (4.09)	9.89 (4.40)	11.01 (4.97)	12.54 (5.56)	<0.001
PHQ-9, M (SD)	5.53 (5.73)	4.06 (4.54)	8.38 (5.61)	10.72 (6.36)	12.54 (7.68)	<0.001
GAD-7, M (SD)	4.23 (5.20)	2.98 (4.15)	6.70 (5.40)	8.63 (6.04)	10.10 (6.90)	<0.001
Total	18,980 (100.0)	14,350 (75.6)	2021 (10.6)	1595 (8.4)	1014 (5.3)	

Note: PHQ-9: the nine-item Patient Health Questionnaire and GAD-7: the seven-item Generalized Anxiety Disorder Scale.

### Proportion of suicide behaviours involvement

Among 18,980 participants, 4630 (24.4%) reported suicide behaviours in the past year. Specifically, 2021 (10.6%) were suicide ideators, 1595 (8.4%) were suicide planners and 1014 (5.3%) were suicide attempters (Table 1). The proportion of suicide behaviour involvement was significantly different by all variables, except for father’s education (*p* = 0.173) and mother’s education (*p* = 0.070). Participants across sex × residence had significantly different proportion of suicide behaviours involvement (Table 2). Particularly, rural females had the highest proportion of suicide ideator (13.8%) and planner (11.5%) according to pairwise comparisons. Besides, scores of five childhood maltreatment subtypes were significantly different between males and females across urban and rural areas.

**Table 2.** The proportion of suicide behaviours and the score difference of childhood maltreatment among participants by sex × residence

	Sex × residence					
Variables	Urban male (*n* = 5116)	Urban female (*n* = 4423)	Rural male (*n* = 4356)	Rural female (*n* = 5085)	*p-*value	Pairwise comparison
Suicide behaviours						
None	4213 (82.3)	3173 (71.7)	3514 (80.7)	3450 (67.8)		
Suicide ideator	385 (7.5)	528 (12.2)	398 (9.1)	700 (13.8)	<0.001	d > b > c > a
Suicide planner	295 (5.8)	414 (9.4)	299 (6.9)	587 (11.5)	<0.001	d > b > c > a
Suicide attempter	223 (4.4)	298 (6.7)	145 (3.3)	348 (6.8)	<0.001	b, d > a > c
Childhood maltreatment						
Physical abuse, M (SD)	5.68 (1.90)	5.56 (1.52)	5.98 (2.13)	5.70 (1.74)	<0.001	c > a, d > b
Emotional abuse, M (SD)	6.23 (2.32)	6.65 (2.59)	6.78 (2.46)	7.20 (2.72)	<0.001	d > b, c > a
Sexual abuse, M (SD)	5.30 (1.51)	5.14 (0.80)	5.55 (1.96)	5.26 (1.20)	<0.001	c > a, d > b
Physical neglect, M (SD)	7.25 (2.82)	6.82 (2.53)	8.09 (3.14)	7.65 (2.91)	<0.001	c > d > a > b
Emotional neglect, M (SD)	8.39 (4.33)	8.82 (4.42)	9.25 (4.68)	9.13 (4.22)	<0.001	c, d > b > a

Note: a, urban male; b, urban female; c, rural male; d, rural female in pairwise comparison.

### Multinomial logistic regression analysis of suicide behaviours involvement

Spearman’s correlations between suicide behaviours and other variables are displayed in Table 3. Results of multinomial logistic regression analysis are shown in Table 4. For total participants, all subtypes of childhood maltreatment were significantly associated with suicide behaviour involvement, except for the ORs between sexual abuse and suicide ideator as well as suicide planner (*p* > 0.05). In subgroup analysis, sexual abuse was significantly associated with suicide attempter for urban males (OR = 1.184, 95% CI 1.121–1.251, *p* < 0.001), urban females (OR = 1.167, 95% CI 1.046–1.302, *p* = 0.006) and rural females (OR = 1.112, 95% CI 1.107–1.197, *p* = 0.010), except for rural males (*p* = 0.084). Physical neglect was significantly associated with suicide ideator only for urban males (OR = 1.041, 95% CI 1.003–1.081, *p* = 0.036). Heat map of associations between childhood maltreatment subtypes and suicide behaviour involvement among participants by sex × residence is depicted in Figure S3 (in Supplementary material).

**Table 3.** Correlations between suicide behaviours and other variables

Variables	1	2	3	4	5	6	7	8	9	10	11	12
1. Suicide behaviours	–											
2. Physical abuse	0.207^***^	–										
3. Emotional abuse	0.346^***^	0.462^***^	–									
4. Sexual abuse	0.116^***^	0.212^***^	0.234^***^	–								
5. Physical neglect	0.176^***^	0.262^***^	0.331^***^	0.156^***^	–							
6. Emotional neglect	0.262^***^	0.329^***^	0.459^***^	0.167^***^	0.561^***^	–						
7. PHQ-9	0.433^***^	0.214^***^	0.412^***^	0.155^***^	0.239^***^	0.318^***^	–					
8. GAD-7	0.415^***^	0.196^***^	0.388^***^	0.150^***^	0.200^***^	0.279^***^	0.827^***^	–				
9. Smoking	0.082^***^	0.067^***^	0.075^***^	0.111^***^	0.081^***^	0.064^***^	0.114^***^	0.099^***^	–			
10. Drinking alcohol	0.121^***^	0.084^***^	0.117^***^	0.120^***^	0.093^***^	0.101^***^	0.148^***^	0.133^***^	0.427^***^	–		
11. Sex	0.139^***^	−0.039^***^	0.113^***^	−0.061^***^	−0.061^***^	0.036^***^	0.164^***^	0.163^***^	−0.095^***^	−0.067^***^	–	
12. Residence	0.040^***^	0.077^***^	0.168^***^	0.079^***^	0.151^***^	0.083^***^	0.255^***^	0.232^***^	0.092^***^	0.042^***^	0.075^***^	–

*** Note:*p* < 0.001.

PHQ-9: the nine-item Patient Health Questionnaire and GAD-7: The seven-item Generalized Anxiety Disorder Scale.

**Table 4.** Multinomial logistic regression of suicide behaviours among participants by sex × residence (OR [95% CI])

Variables	Suicide ideator	*p-*value	Suicide planner	*p-*value	Suicide attempter	*p-*value
Model 1 (total)						
Physical abuse	1.077 (1.049−1.107)	<0.001	1.134 (1.105−1.164)	<0.001	1.219 (1.186−1.252)	<0.001
Emotional abuse	1.150 (1.127−1.172)	<0.001	1.202 (1.178−1.226)	<0.001	1.271 (1.243−1.300)	<0.001
Sexual abuse	1.005 (0.969−1.043)	0.786	1.007 (0.968−1.048)	0.720	1.118 (1.081−1.157)	<0.001
Physical neglect	1.024 (1.006−1.042)	0.009	1.070 (1.050−1.090)	<0.001	1.137 (1.112−1.162)	<0.001
Emotional neglect	1.051 (1.039−1.063)	<0.001	1.079 (1.066−1.092)	<0.001	1.119 (1.103−1.135)	<0.001
Model 2 (urban male)						
Physical abuse	1.072 (1.016−1.130)	0.010	1.119 (1.063−1.179)	<0.001	1.186 (1.127−1.248)	<0.001
Emotional abuse	1.164 (1.116−1.215)	<0.001	1.210 (1.158−1 265)	<0.001	1.284 (1.227−1.344)	<0.001
Sexual abuse	0.921 (0.825−1.028)	0.142	1.025 (0.945−1.111)	0.555	1.184 (1.121−1.251)	<0.001
Physical neglect	1.041 (1.003−1.081)	0.036	1.062 (1.018−1.107)	0.005	1.180 (1.131−1.231)	<0.001
Emotional neglect	1.035 (1.010−1.061)	0.006	1.077 (1.050−1.106)	<0.001	1.115 (1.085−1.147)	<0.001
Model 3 (urban female)						
Physical abuse	1.112 (1.037−1.192)	0.003	1.217 (1.139−1.301)	<0.001	1.343 (1.256−1.436)	<0.001
Emotional abuse	1.170 (1.123−1.220)	<0.001	1.262 (1.210−1.317)	<0.001	1.320 (1.260−1.382)	<0.001
Sexual abuse	0.945 (0.819−1.091)	0.439	0.950 (0.811−1.112)	0.521	1.167 (1.046−1.302)	0.006
Physical neglect	1.033 (0.992−1.076)	0.115	1.107 (1.061−1.154)	<0.001	1.115 (1.063−1.170)	<0.001
Emotional neglect	1.084 (1.059−1.108)	<0.001	1.104 (1.076−1.132)	<0.001	1.132 (1.100−1.165)	<0.001
Model 4 (rural male)						
Physical abuse	1.071 (1.023−1.121)	0.004	1.074 (1.022−1.129)	0.005	1.170 (1.108−1.235)	<0.001
Emotional abuse	1.133 (1.008−1.180)	<0.001	1.145 (1.095−1.196)	<0.001	1.244 (1.180−1.312)	<0.001
Sexual abuse	1.021 (0.972−1.072)	0.411	0.996 (0.939−1.057)	0.894	1.058 (0.992−1.128)	0.084
Physical neglect	1.017 (0.983−1.052)	0.337	1.018 (0.980−1.059)	0.360	1.093 (1.039−1.149)	0.001
Emotional neglect	1.057 (1.034−1.081)	<0.001	1.050 (1.023−1.077)	<0.001	1.101 (1.065−1.139)	<0.001
Model 5 (rural female)						
Physical abuse	1.075 (1.017−1.136)	0.010	1.164 (1.105−1.226)	<0.001	1.264 (1.195−1.338)	<0.001
Emotional abuse	1.139 (1.100−1.178)	<0.001	1.184 (1.143−1.226)	<0.001	1.251 (1.201−1.303)	<0.001
Sexual abuse	1.030 (0.955−1.112)	0.442	1.016 (0.935−1.103)	0.715	1.112 (1.026−1.206)	0.010
Physical neglect	1.016 (0.984−1.048)	0.332	1.087 (1.052−1.122)	<0.001	1.151 (1.107−1.197)	<0.001
Emotional neglect	1.031 (1.010−1.053)	0.004	1.082 (1.059−1.105)	<0.001	1.125 (1.095−1.155)	<0.001

Note: Model 1 was adjusted for sex, residence, age, grade, family composition, family income, smoking, drinking alcohol, depression and anxiety. Model 2 to Model 5 were adjusted for age, grade, family composition, family income, smoking, drinking alcohol, PHQ-9 score and GAD-7 score. Each subtype of childhood maltreatment was the only independent variable entered into the models.

### Structural equation modelling for suicide behaviours

Figure S4 (in Supplementary material) showed the results of structural equation modelling. After controlling for covariates and the interaction between five subtypes of childhood maltreatment, the direct effects of childhood maltreatment subtypes on suicide behaviours from high to low were emotional abuse (*β* = 0.363, *p* < 0.001), physical abuse (*β* = 0.100, *p* < 0.001) and sexual abuse (*β* = 0.033, *p* = 0.003). However, the effects of physical neglect (*β* = 0.036, *p* = 0.507) and emotional neglect (*β* = 0.029, *p* = 0.587) on suicide behaviours were not significant.

## Discussion

This is the first study to quantify the associations between five core subtypes of childhood maltreatment and three stages of suicide behaviours among Chinese adolescents. The current study has several key and new findings. First, different subtypes of childhood maltreatment have specific and unique associations with suicide behaviours. For example, sexual abuse is significantly related to suicide attempter but is not significantly related for suicide ideator and planner. This result may reveal an acute effect of sexual abuse on suicidality. Second, the associations between childhood maltreatment subtypes and suicide behaviours differ between males and females across urban and rural China. Third, the direct effects of childhood maltreatment subtypes on suicide behaviours are non-equivalence. Specifically, emotional abuse has the greatest effect, while the effects of physical and emotional neglect are not significant. These findings could benefit for policy-makers in preventing suicidality for Chinese adolescents with or without childhood maltreatment experiences.

Our results demonstrate that the associations between different childhood maltreatment subtypes and suicide behaviour involvement vary considerably, which is congruent, with some existing literature concluded that various forms of childhood maltreatment maintain a unique and independent influence on suicidal risk (Angelakis *et al.*, 2020; Zatti *et al.*, 2017). It might be possible that different subtypes of childhood maltreatment evoke the feeling of constraint and desperation in a different way (Mcmahon *et al.*, 2018; O’Connor and Portzky, 2018). On contrary, some prior literature shows that all subtypes of childhood maltreatment have a similar or equivalent effect on suicide behaviours (Guo *et al.*, 2018; Hoertel *et al.*, 2015). The inconsistency may stem from different study designs (prospective or cross-sectional), sampling methods (random or convenient) and mixed samples (adolescents or adults, community or clinical samples) (Miché *et al.*, 2020). On the other hand, the definition and measurement of childhood maltreatment are varied across previous studies (Grillault *et al.*, 2022; Miller *et al.*, 2013; Park *et al.*, 2020). The most widely used assessment of five childhood maltreatment subtypes is the CTQ-SF (Bernstein *et al.*, 2003). However, some scholars treat subtypes of childhood maltreatment as five dichotomous variables (Ahouanse *et al.*, 2022; Chen *et al.*, 2021b; Xie *et al.*, 2018), while others treat them as five continuous variables (Gong *et al.*, 2020; Guo *et al.*, 2018), which could result in heterogeneity across the existing studies.

Some previous studies believe that sexual abuse has a stronger effect on suicidality than other subtypes (Angelakis *et al.*, 2020; Mcmahon *et al.*, 2018). However, after considering the potential multicollinearity among five subtypes of childhood maltreatment, our results indicate that emotional abuse might have the most harmful effect on suicide behaviours. The difference may be related to the cultural and social background between China and other countries (Stoltenborgh *et al.*, 2011). Traditional Chinese culture, such as Confucian values, strongly inhibits the disclosure of sexual abuse experiences, especially for adolescents (Ji *et al.*, 2013). On the other hand, compared with physical and sexual abuse, emotional abuse might be hard to detect because of less obvious signs. However, emotional abuse in adolescents can lead to shame, hopelessness, avoidance and a dysfunctional attachment pattern, which will increase the risk of depression and anxiety. These two psychological disorders are well-recognized risk factors of suicidality (Chen *et al.*, 2021b; Lee *et al.*, 2018). In addition, some specific behaviours of emotional abuse, such as parental rejection and low parental warmth or responsiveness, may lead to low self-esteem, a negative coping style and a desperate worldview (Lee *et al.*, 2021). Thus, emotional abuse could be a strong predictor of internal psychopathology development and might disturb the psychosocial well-being during childhood and adolescence (Ahouanse *et al.*, 2022).

Notably, we found that sexual abuse is only significantly related to suicide attempter but not for suicide ideator and planner. However, other four subtypes of childhood maltreatment are concurrently associated with suicide ideator, planner and attempter. This finding may mean that the effect of sexual abuse on suicide behaviours is acute and is different between other childhood maltreatment forms. For victims of sexual abuse, they may have suicide ideation, plans and attempts simultaneously in a short period of time and become a suicide attempter. Therefore, they are less likely to be a suicide ideator or planer. The innovative finding should be confirmed with more follow-up studies (Lee *et al.*, 2021). Regarding sex difference, some previous studies believe that the associations between childhood maltreatment subtypes and suicidality differ between males and females (Liu *et al.*, 2017; Mcmahon *et al.*, 2018; Miché *et al.*, 2020; Salokangas *et al.*, 2019). However, to our knowledge, little is known that whether the associations differ between urban and rural adolescents. In the current study, it is interesting to note that the proportion of suicide behaviour involvement is significantly different between males and females across urban and rural China. Notably, rural females may be the most vulnerable population of suicidality. Therefore, suicide prevention programs could give priority to female adolescents in rural China.

### Limitations

Several limitations should be acknowledged. First, the cross-sectional design of the current study does not allow us to draw any causal relationships between childhood maltreatment subtypes and suicide behaviours. Future studies could benefit from a longitudinal design. Second, the self-reported questionnaire in the current study has some sensitive questions for Chinese adolescents. Participants who submitted incomplete questionnaires without reports of suicide behaviours or (and) childhood maltreatment, especially for sexual abuse, may be more likely to suffer suicide risk. Future research could take multi-informant measures to obtain these sensitive information, such as prospective caregiver reports (usually from the mother) (Newbury *et al.*, 2018). Third, other important information of childhood maltreatment was not evaluated, such as the age of the first maltreatment experience, duration, intensity, frequency and perpetrator of abuse or neglect (Grillault *et al.*, 2022). In the next step, we will conduct a survey to collect more related information to further explore the impact of timing, duration, severity and offender of childhood maltreatment (Duarte *et al.*, 2020). Finally, although the study highlights independent and specific effects of different childhood maltreatment subtypes, the co-occurrence of five subtypes is ignored. In future research, a latent class (profile) analysis should be used to examine the relationship between co-occurrence of multiple childhood maltreatment subtypes and suicidality (Luk *et al.*, 2021; Witt *et al.*, 2016).

### Implications

A better understanding of the associations between different childhood maltreatment subtypes and suicide behaviours could advance the development of efficient preventive strategies. Our findings have some practical implications for Chinese adolescents. First, given that not all childhood maltreatment subtypes is significantly associated with suicide ideator, planner and attempter. The finding emphasizes the importance of differentiating the specificity of different forms of childhood maltreatment, which can help decision-maker to develop a tailored preventive approach for suicidality. For example, sexual abuse is a risk factor of suicide attempter but not for suicide ideator and planner. We should take immediate measures for those who have history of sexual abuse to keep them away from dangerous stuffs, such as knives and sleeping pills. Then, more efforts could be taken to reduce their chances of being suicide attempter. Second, emotional abuse may be the strongest predictor of suicide behaviours in five subtypes, while the effects of physical neglect and emotional neglect are not significant. Therefore, suicide interventions could focus on those who have history of emotional abuse. Increasing parental support for children and positive interaction with offspring will be a promising approach to weaken the effect of emotional abuse and prevent suicide behaviours. Third, the associations between childhood maltreatment subtypes and suicide behaviours differ by sex and residence. This finding may encourage health professionals to implement unique strategies between males and females across urban and rural China. For instance, among rural males, we could pay more attention to those who experienced physical abuse, emotional abuse and emotional neglect rather than sexual abuse when we only have limited resources for suicide intervention.

## Conclusions

Five core subtypes of childhood maltreatment have specific and non-equivalence associations with suicide ideator, planner and attempter in Chinese adolescents. The finding highlights the specificity and severity of different childhood maltreatment forms on suicide behaviours. Emotional abuse may have the strongest effect and sexual abuse have an acute effect on suicide behaviours. Suicide prevention programs could focus on those who experienced emotional, physical and sexual abuse. Furthermore, the associations between childhood maltreatment subtypes and suicide behaviours differ between males and females across urban and rural China. Intervention strategies of suicide should be tailored by sex and residence. Particularly, rural females may be the most vulnerable population of suicidality, and they deserve more attention.

## Data Availability

The datasets used and/or analysed during the current study are available from the corresponding author on reasonable request.

## Supplementary material

For supplementary material accompanying this paper visit http://doi.org/10.1017/S2045796023000604. click here to view supplementary material
