# Supplementary material for: Specific effects of five subtypes of childhood maltreatment on suicide behaviours in Chinese adolescents: the moderating effect of sex and residence
Source: Epidemiol Psychiatr Sci. 2023 Jul 11;32:e45. doi: 10.1017/S2045796023000604 (PMC10387488; doi:10.1017/S2045796023000604)
Supplement: Supplementary file 1 [file S2045796023000604sup001.docx]

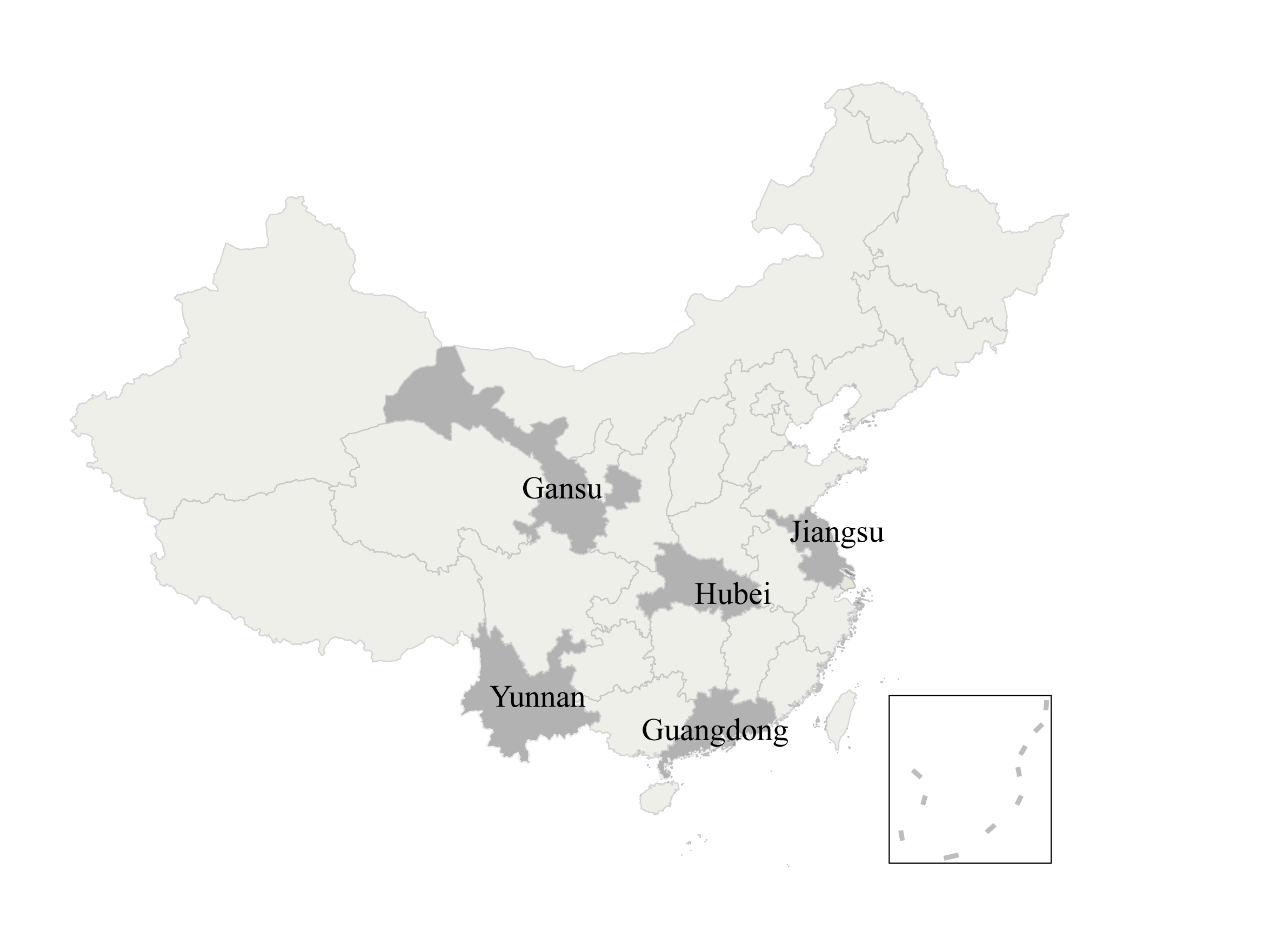


**Figure 1.** Five selected provinces from the eastern, southern, western, northern, and central China.

**
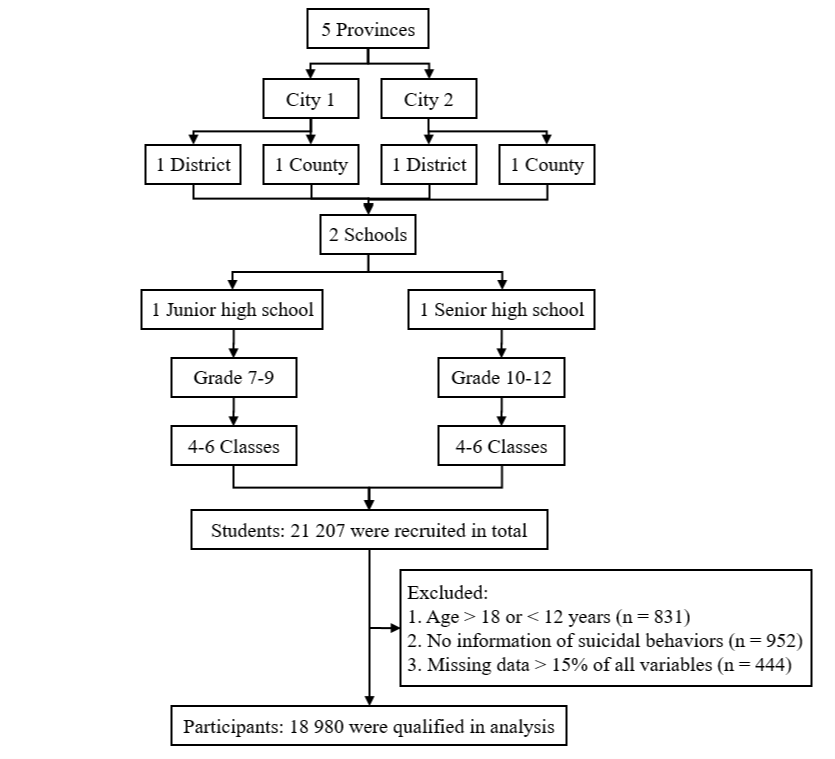
**

**Figure 2.** The process for multi-stage cluster sampling and study participants selection.

**
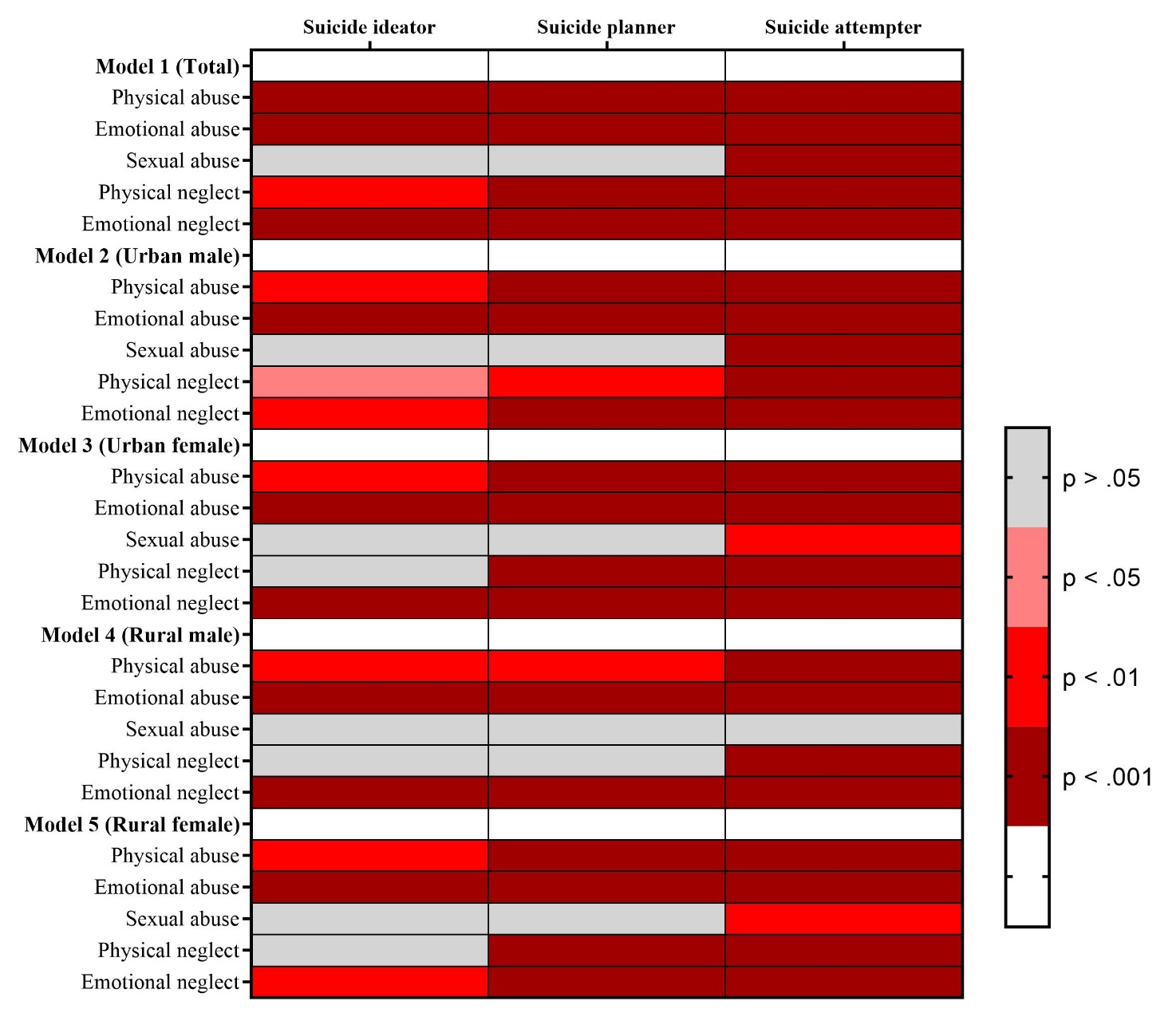
**

**Figure 3.** Heat map of associations between childhood maltreatment subtypes and suicide behaviors involvement among participants by Sex × Residence (based on *p*-values).

Note. All models were adjusted for age, grade, family composition, family income, smoking, drinking alcohol, PHQ-9 score and GAD-7 score.


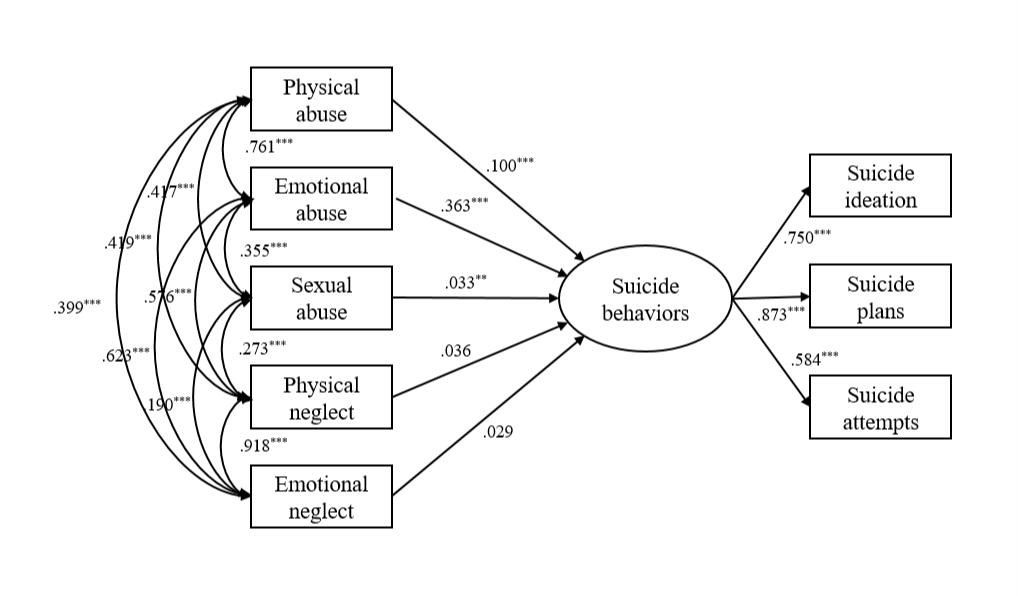


**Figure 4.** Structural equation modeling predicting suicide behaviors from five subtypes of childhood maltreatment after controlling for covariates.

Note. Covariates including sex, residence, age, grade, family composition, family income, smoking, drinking alcohol, PHQ-9 score and GAD-7 score.

^**^ *p* < 0.01, ^***^ *p* < 0.001.
